# The case for cautious paternalism in the emergency management of patients with borderline personality disorder

**DOI:** 10.1192/bjb.2020.148

**Published:** 2021-04

**Authors:** Giles Newton-Howes

**Affiliations:** University of Otago, New Zealand

**Keywords:** Borderline personality disorder, capacity, consent, affect dysregulation, autonomy

## Abstract

Principlism is the dominant ethical theory in modern medicine. Autonomy is ‘king’ of the principles espoused and operationalised in consent. Consent is the mechanism by which all medical interactions occur. In borderline personality disorder (BPD) there is often a diffuse sense of self, emotional instability and impulsivity that can lead to medically dangerous non-suicidal self-injury, acute medical intervention and then a withdrawal of consent while the potential threat to the person's well-being remains high. Claims of lack of capacity lack veracity, and simply acting against the patient's will may be illegal. Understanding the will and preferences of patients is a step forward, but it is not always possible in time-sensitive situations. A cautious paternalism is therefore warranted both to ensure the patient's well-being while being honest as to the reasons for this, and to possibly build epistemic trust between the medical system and the patient with BPD.

Principlism has been the dominant force in medical ethics for more than half a century.^1^ Its four principles – autonomy, beneficence, non-maleficence and justice – form a common touchstone in medicine, particularly when hard choices need to be made.^2^ Although neither Beauchamp nor Childress, the authors of this bioethical approach, weighted any one of these principles as more important than any other, autonomy has risen as the prominent principle.^3^ This is not surprising, as autonomy is operationalised into informed consent, the tool doctors use to assess capacity and then engage in clinical action.^4^ Capacity assessment based on principlism is purely process driven, based on whether a patient understands, retains, weighs and answers questions. The content of the decision is explicitly ignored. Where doctor and patient agree, or where the patient agrees with the doctor, an assessment of autonomy through capacity is rarely detailed. There is little need, as all parties agree as to the best approach to take. This is not true, however, when the patient disagrees with the doctor. In these cases, the medical mind is more likely to focus on the issue of capacity, as if it is not present a ‘duty of care’ may suggest a course of action that contravenes the patient's explicitly stated desires.^5^ Most conventional wisdom and practice dictate that a loss of capacity is, largely, the only legitimate rationale for acting in such a way, and to act differently requires confirmation by the court. Specific mental health legislation is a glaring exception, and efforts to restrict its use or abolish this legislation are occurring in multiple jurisdictions (for example, the new fusion legislation of Northern Ireland). As the risks of a poor outcome increase, so does the doctor's desire to act differently from the patient's stated desire, if her desire may have a possible catastrophic outcome. Even if this is the case, the court may honour the patients' autonomy, despite a guaranteed outcome of death. Put simply, autonomy is king, and to dethrone the king is socially and legally increasingly less acceptable. The question is whether this approach is the best one for patients with borderline personality disorder (BPD).

## The challenge of BPD

BPD is characterised by a diffuse sense of self, difficulty in understanding affect, intense unstable interpersonal relationships and impulsivity.^6^ These features of the disorder are pervasive, reaching into all areas of a person's life. They commonly, but not exclusively, arise in adolescence and cause considerable morbidity to a young population.^7^ Health services are often engaged in the management of patients with BPD, as they are treatment-seeking, recognising their emotional and interactive difficulties and experiencing significant distress because of them. Psychotherapeutic approaches support long-term improvement; however, personal responses to short-term distress often include thoughts of suicide and non-suicidal self-injury (NSSI). Both expressions of suicide and NSSI cause significant distress to healthcare workers inside and outside mental health and can be difficult to manage. For actions that lead to self-harm, it can be difficult to ascertain whether the antecedent to the action is an effort to end life, or some other cause such as to regulate emotional distress or ‘feel something’. If the purpose of NSSI is to regulate emotion, then the support and investigation of emergency services may provide sufficient supportive human contact to allow the emotional storm to pass. Now the patient sees no need for treatment, and this can be very distressing to medical staff, particularly if the NSSI is potentially lethal. Life-threatening overdose is an excellent example of this. In these circumstances, medical staff can see the potential danger of non-adherence to emergency treatment, whereas the patient, with a clear sensorium and who clearly understands the information provided, may still demand to go home as their immediate emotional needs have been met.

## The clinical conundrum

This is a clinical conundrum: in observing the patient's autonomy, one appears ethically obliged to do what may be (or certainly is) life-threatening. Disregarding the patient's autonomy to preserve life appears ethically dubious, as autonomy is after all king.

## Questioning capacity

In such cases, the default position has largely been to question capacity. This is internally consistent with the bioethics of principlism, which is built on a normative moral philosophy. Such normative moralities are not dependent on a series of complex ethical foundations, rather, central to their claim is the idea that the morality of a society's members, common sense and tradition are its basis (see Ref. 2, pp. 3–10, where the notion of a ‘common morality’ is spelt out). Principlism is, however, in direct conflict with the growth of a human rights ethic, which values autonomy as separate from any such normative requirement. Indeed, this human rights ethic challenges the normative position of enabling one person to do something to another without their consent. This recognises the increasingly diverse components of society and the need to ensure all have equal rights. The Convention on the Rights of Persons with Disabilities (CRPD)^8^ is the clearest example of this. The CRPD, of which most nation states are signatories, requires signatories to uphold the equal rights of those with disabilities, including those with mental health conditions, as being equal to those without such disorders. The basis of this is a social model of disability.^9^ The social model directly challenges the normative tradition of our bioethics, on which ethical clinical practice is based. It suggests that a key problem with the medical model is the way society limits freedom and choices. For example, a medical model states that those who cannot see should not drive. This seems obvious and incontrovertible. The social model of disability challenges this to say that those who cannot see should be allowed to drive, in cars appropriately equipped to ensure they and others are safe, for instance, self-navigating cars. This combination of a human rights ethic and the social model of disability challenges the ethical validity of principlism, its common morality.

In the clinical conundrum described above, the appeal to a failure of capacity usually relates to an inability to weigh the evidence in the balance (by way of process). On these grounds, capacity – and ergo autonomy – fails, and the doctor has a duty of care to step in. This stance is not the case for many people with BPD, who largely retain this ability. This means that preventing a patient with BPD from leaving hospital, as described above, breaches their human right to autonomy. This ‘medical model’ approach is also challenged by the social model of disability. This model places the need for care in society (for example, by providing care at home) and would not support retaining someone in hospital despite the potentially life-threatening consequences of leaving. There are, therefore, no ethical grounds to act in such a way using either a principlist or a human rights ethic. So, if patients with BPD can make cross-sectional autonomous choices that may have catastrophic consequences, should they be freely allowed to do so?

## The third way: will and preferences

Using a purely process approach to capacity clearly fails to ensure the well-being of a patient with BPD in a situation such as a life-threatening overdose if she refuses medical treatment. Simply withdrawing treatment may also fail to ensure the well-being of patients with BPD in the same circumstances. Are clinical staff doomed to fail such patients? The answer to this seems to lie in a clearer consideration of the notion of autonomy. Regardless of whether autonomy is normative (as the bioethicists purport) or idiosyncratic (as the human rights proponents purport), it implies a sense of self, a notion of understanding ‘who I am and what I want’. Further, it implies a sense of continuity to these expressed desires, desires that could be reasonably expected to change only slowly over time. Without these two implied requirements, a clear sense of self and a temporal stability to this sense of self, both idiosyncratic and normative judgement become mere chaos, a changeable noise without foundation. This identifies a third way forward: rather than focusing on the capacity process, an understanding of the content of the decision and the context of the person may allow a more nuanced understanding of autonomy and, subsequently, capacity. The focus then shifts from a response to the cross-sectional assessment of how you are now to an assessment of what you may want across time. Such a shift necessarily requires consideration of the content of the decision, as well as the process of decision-making. This has been described as weighing an individual's will and preferences,^10^ as opposed to focusing on the process of decision-making in capacity or a cross-sectional assessment of this decision at this time. This appears to be a more authentic form of considering decision-making capacity.^11^ In the case of the patient who has taken a life-threatening overdose, this might lead to a completely different decision. Rather than allowing such a person to leave the emergency department, considering their process of decision-making to be intact, they may be kept to ensure their safety based on their longer-term will and preferences (for example, plans for the future). This move towards will and preferences-based decision-making in medicine appears to be a significant step forward. It recognises the individual and idiosyncratic rights of the person. It supports the person to make the best choices for them in their context, and enables the medical system to be clear that it is doing the best for the patient, not simply leaving them to their fate. Although only required in cases of disagreement, this process is likely to be lengthy, and requires information from collateral sources and repeated interviews to develop such a nuanced sense of will and preferences.

## The case for a cautious paternalism

None of this is likely to be possible in emergency settings where decisions are time critical. Added to this in people with BPD is a diffuse sense of self, a core characteristic. Such a clear understanding of will and preferences is likely to be an order of magnitude more difficult to understand in such circumstances. The patient herself may not understand her own drives or why life is so emotionally and interpersonally distressing. In these circumstances, the reality of clarifying will and preferences in an appropriate and consistent manner is very unlikely. Rather than defaulting to an obviously flawed argument to suggest the patient lacks capacity, a cautious paternalism may be more honest, supportive and applicable. Despite some of the difficulties for people with BPD in general, a sense of understanding others is clear, and using a flawed system, in which they are considered to lack capacity, simply increases distrust in the medical system and medical model of care. Cautious paternalism recognises the difficulties of a consistent sense of self for people with BPD and of understanding their will and preferences in a timely manner. It does not rely on a tautological suggestion of a lack of capacity for patients who disagree with medical decisions (because you disagree with me, you *ipso facto* lack capacity). Rather, it provides medical support for well-being and potentially increases epistemic trust^12^ between the patient and the medical system, a trust that people with BPD have significant problems with. This is likely to require the time and skill to engage with the patient to persuade them of the need for review and reassure them of the intent to support autonomy while at the same time recognising the limits to this inherent in their presentation. If they leave, then accessing support (for example, via family or police) to return them to the emergency department recognises the limits to autonomy implied by this presentation and prevents possible ill-considered catastrophic harm.

Such a cautious paternalism may help to find a way forward that enables people with BPD to both receive adequate medical care in emergencies and develop trust in a system with the possibility of longer-term therapeutic support.

## About the author

**Giles Newton-Howes** is an Associate Professor at the University of Otago, Wellington, New Zealand.
